# Environmental and occupational respiratory diseases – 1034. A novel ELISA method for the detection of dust mite allergens using IgY technology

**DOI:** 10.1186/1939-4551-6-S1-P33

**Published:** 2013-04-23

**Authors:** Dary Luz Mendoza, Gloria Garavito, Luis Alejandro Barrera, Angela Espejo, Elkin Navarro, Eduardo Egea

**Affiliations:** 1 Ciencias Basicas, Universidad Del Atlantico, Colombia; 2 División Ciencias De La Salud, Universidad Del Norte, Barranquilla, Colombia; 3 Pontificia Universidad Javeriana, Colombia

## Background

One of the main strategies to reduce the expression of allergic symptoms in atopic patients sensitized to house dust mites is the use of detection system for monitoring and reduce environmental allergens in indoor spaces. In this context the IgY technology represent a valuable tool. The specific aims of this study was to standardize a *in house *method of indirect ELISA assay for detection of mite allergens using mite IgY antibodies.

## Methods

We used two specific avian antibodies against synthetic oligopéptidos raised and designed from the mature cistein protease that previously were development by us: anti-P04 and anti-P06. The specificity of this IgY were evaluated using Dermatophagoides farinae(Df) and Blomia tropicalis(Bt) and Periplaneta americana(PA) extracts. Dust samples were taken from mattresses and it were collected in cellulose filters, then extracted and diluted in 1X PBS, pH 7.4. Antibodies was diluted 1/250. Dust samples from unused brand new mattresses **were** used as negative control.

## Results

Both IgYs, anti-P04 and anti-P06, showed reactivity against Df and Bt extracts, but not against the extract of P A. The anti-P04 IgY showed higher sensitivity compared to the IgY anti-P06. The limit detection at the mite extracts was > 0.3 ug / mL protein. Antibodies were able to detect allergens at a Limiting dilution of the dust samples until 1/8 for igY anti P04-and ¼ for anti-IgY PO6. Samples tested by ELISA using dust from unused mattresses were negative. Direct relationship was observed between the number of Dermatophagoides sp in the sample when it was comparative with OD of the ELISA assay.

## Conclusions

Specific IgY antibodies seems to be useful for the detection of mites allergens in dust samples. This *in house* indirect ELISA could be used routinely in the laboratory for detection of mite allergens in indoor spaces. This study provides the basis for the detection and monitoring of other allergens intradomiciliary.

